# Extinction and Renewal of Conditioned Sexual Responses

**DOI:** 10.1371/journal.pone.0105955

**Published:** 2014-08-29

**Authors:** Mirte Brom, Ellen Laan, Walter Everaerd, Philip Spinhoven, Stephanie Both

**Affiliations:** 1 Institute of Psychology, Clinical Psychology Unit, Leiden University, The Netherlands; 2 Department of Psychosomatic Gynaecology and Sexology, Leiden University Medical Centre, Leiden, The Netherlands; 3 Department of Sexology and Psychosomatic Obstetrics and Gynaecology, Academic Medical Centre, University of Amsterdam, Amsterdam, The Netherlands; 4 Department of Clinical Psychology, University of Amsterdam, Amsterdam, The Netherlands; 5 Department of Psychiatry, Leiden University Medical Centre, The Netherlands; Technion - Israel Institute of Technology, Israel

## Abstract

**Introduction:**

Extinction involves an inhibitory form of new learning that is highly dependent on the context for expression. This is supported by phenomena such as renewal and spontaneous recovery, which may help explain the persistence of appetitive behavior, and related problems such as addictions. Research on these phenomena in the sexual domain is lacking, where it may help to explain the persistence of learned sexual responses.

**Method:**

Men (n = 40) and women (n = 62) participated in a differential conditioning paradigm, with genital vibrotactile stimulation as US and neutral pictures as conditional stimuli (CSs). Dependent variables were genital and subjective sexual arousal, affect, US expectancy, and approach and avoid tendencies towards the CSs. Extinction and renewal of conditioned sexual responses were studied by context manipulation (AAA vs. ABA condition).

**Results:**

No renewal effect of genital conditioned responding could be detected, but an obvious recovery of US expectancy following a context change after extinction (ABA) was demonstrated. Additionally, women demonstrated recovery of subjective affect and subjective sexual arousal. Participants in the ABA demonstrated more approach biases towards stimuli.

**Conclusions:**

The findings support the context dependency of extinction and renewal of conditioned sexual responses in humans. This knowledge may have implications for the treatment of disturbances in sexual appetitive responses such as hypo- and hypersexuality.

## Introduction

It is thought that contexts play an important role in regulating responses and in related relapse behavior [1], [2]. The role of context is best exemplified by the phenomenon of renewal. Renewal is the term used to describe recovery of extinguished behavior as a result of context change [3]. The renewal phenomenon has been demonstrated for Pavlovian and instrumental responding based on numerous reinforcers, including natural rewards such as food [4] and drug rewards [5]. Unfortunately, given its relevance for extinction-based treatments, studies on extinction and renewal in the human sexual domain are completely lacking. In the present paper, we report an experiment on extinction and renewal of conditioned sexual responses in sexually functional men and women.

According to incentive motivation models, sexual motivation is the result of the interplay of a sensitive internal sexual system with motivational stimuli. Stimuli that can promote motivation are called incentive stimuli [6], [7]. Their motivational valence can be unconditioned or conditioned as a result of associative leaning [8]. Some stimuli (e.g. genital touch) may be innately sexually competent (i.e. stimuli that can elicit sexual response without a learning history) and can therefore serve as incentive stimuli, but many sexual stimuli are not intrinsically sexually competent. Specific cues of sexually competent stimuli may gain learned incentive value through their association with the stimulus. Several studies, including some from our lab, have demonstrated conditioned sexual arousal responses in humans [9]. In classical conditioning, through the repeated association of a neutral stimulus (NS) with the unconditional stimulus (US), the NS will eventually trigger the same reaction as the US. Since the NS is no longer ineffective in eliciting a response but has become a conditioned stimulus (CS), the reaction to the CS is called the conditioned response (CR) [6], [10]. Subsequent repeated presentations of a CS without the US will result in a loss of conditioned responding, as the CS no longer predicts the aversive or appetitive US [11]. This learning process is known as *extinction* and has obvious clinical relevance as it is thought to be the core mechanism for exposure therapy [12]–[14]. In exposure therapy, conditioned responses are lessened or inhibited by repeated or prolonged exposure to a cue (the CS) in absence of the event it used to predict (the US). However, many individuals relapse after being ‘cured’. Therefore, although CS-alone presentations may extinguish conditioned responses, the extinction procedure does not seem to erase the originally learned CS-US association. It appears that this original association is retained [3].

Conditioned responding can ‘renew’ following a context shift out of the extinction context [1]. Renewal is the restoration of the CR in context A but not in context B when learning occurred in context A and extinction in context B. Extrapolating the renewal phenomenon to clinical practice, someone who acquired craving for internet-sex at home (context A), and is successfully extinguished by cue exposure therapy in a therapeutic setting (context B), may experience strong craving upon changing context such as sitting behind the computer at home (context A).

The assumption that conditioned responses extinguish dependent upon context has been supported by animal studies [15]. In humans, the phenomenon of renewal is mainly studied in fear paradigms or studies on addiction [2], [16]–[18]. It is demonstrated that fear returns when individuals are tested in a context different from the treatment context [12]. In a differential fear conditioning experiment by Vansteenwegen et al. [19], a neutral slide of a pictorial face (CS+) was paired with a loud noise (US). The CS+ is the stimulus that is being followed by the US, whereas the CS− is not. Extinction of conditioned fear was established by presenting the CS without the US in a different context. Different contexts were obtained by manipulating the lighting in the experimental room, and acquisition took place in either a dark or illuminated room. When returning to the original acquisition context (i.e., ABA renewal), conditioned fear responding to the CS+ renewed. Effting and Kindt [16] replicated this renewal effect in humans within an ABA renewal paradigm as used by Vansteenwegen et al. [19], making use of shocks as US. Changing the context after the extinction phase resulted in a significant increase of US expectancy ratings to CS+ relative to CS− in Context A. However, no robust renewal effect for electrodermal responses could be demonstrated. In addition, there is evidence for renewal of conditioned responses following a context change in appetitive conditioning [2],[20].

Although the evidence regarding renewal in human learning has accumulated in recent years, studies on renewal of sexual conditioned responses are lacking, despite the possible important implications for exposure-based treatment strategies for learned maladaptive sexual responses. The finding that paraphilia, hypersexuality and related sexual disorders are predominantly observed in men [21], [22] has led to the idea that men are more receptive to sexual conditioning than women, resulting in increased CR acquisition [23]. However, at present, it is not clear if gender differences in sexual conditionability do exist as results of conditioning studies are mixed [24]–[26]. However, a vast amount of research has shown that the neurotransmitter dopamine plays a major role in associative learning and sexual behaviour [27]–[29], and as gender differences in the number of dopamine neurons are influenced by several factors, including sex chromosome complement [30], the presence of the Sry gene [31] and gonadal hormones, it is therefore thinkable that differences in sexual conditionability do exist between men and women, with men being more receptive to sexual conditioning. This, combined with the fact that adolescent maturation is a sensitive period for steroid dependent organization of neural circuits involved in sexual stimulus salience, sensory associations and sexual motivation [32], and the finding that for men, more than for women, visual stimuli preferentially recruit an amygdalo-hypothalamic pathway [33], gender differences in sexual conditionability seem plausible. In addition, it is proposed that sexual preferences of men are greatly influenced by early learning experiences, particularly during defined critical periods, such as adolescence [23], [34]. In addition, women are believed to have more ‘erotic plasticity’ [35]. The contradictory results of previous studies point to the importance for further investigation of possible gender differences in sexual learning.

The present study is the first to investigate extinction and renewal of conditioned sexual responses in men and women. The experimental procedure of Effting and Kindt [16] was closely followed, except that now a sexually pleasurable tactile stimulus (vibrotactile genital stimulation) served as US. Two neutral pictures served as CSs, and subjective affect, subjective sexual arousal, US expectancy ratings and genital arousal were dependent variables. It was predicted that participants in both conditions (AAA and ABA) would show conditioned sexual responding after acquisition trials, which was expected to gradually decrease. As an index of renewal, it was predicted that upon a context change after extinction, the ABA condition would show recovery of conditioned responding on the test trials as compared to the last extinction trial. No increases were expected during these test trials in the AAA condition. In addition a stimulus response compatibility task (Approach and Avoidance Task, AAT) was included to assess implicit approach and avoidance tendencies towards the CS [36]. It was predicted that upon a context change after extinction, participants in the ABA condition would show stronger approach responding to CS+ relative to CS− on the AAT as compared to participants within the AAA condition.

## Method

### Participants

Written consent was obtained from all participants before participation. Research participants were 40 men and 62 women. Participants were paid (€30,−) or received course credit for their participation. Participants were recruited through advertisements on social networks, and at the Universities of Leiden and Amsterdam. Inclusion criteria were: age between 18 – 45 years and a heterosexual orientation. Exclusion criteria were: sexual problems, an affective or psychotic disorder or abusive drug use, pregnancy or breastfeeding, and a medical illness or medication use that could interfere with sexual response. The study was approved by the Medical Ethical Committee of the Leiden University Medical Center.

### Design and conditioning procedure

**Figure 1:**
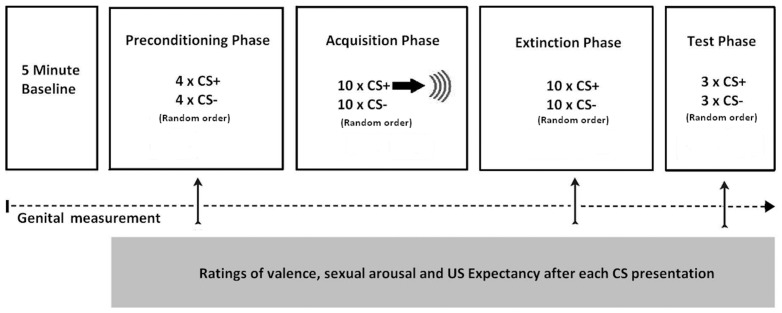
Schematic representation of the experimental procedure in both context conditions. In the AAA-condition, acquisition phase, extinction phase and test phase were in the same lighting context. In the ABA-condition the extinction phase was in a different lighting context than the acquisition phase and test phase.

The experimental design involved differential conditioning with one stimulus (the CS+) being followed by genital vibrostimulation (US) during the acquisition phase, whereas the other stimulus (CS−) was never followed by genital vibrostimulation. Which of the two stimuli served as the CS+ was counterbalanced across participants and conditions. In the ABA condition, participants received acquisition in one context (Context A), extinction in another context (Context B), and a test for renewal in the original acquisition context (Context A). In the AAA condition, acquisition, extinction, and testing took place in one and the same context (Context A). The colors of the lighting that served as Contexts A and B were counterbalanced across participants. For a schematic overview of the procedure see Figure 1.

In the preconditioning phase, participants saw four nonreinforced presentations of the CS+ and four presentations of the CS−. Subsequently, in the acquisition phase the contingency between CS+ and US was learned. During this phase both the CS+ and CS− were presented 10 times each and the CS+ was always followed by the US. For participants in all conditions acquisition took place in Context A. The extinction phase consisted of 10 unreinforced CS+ presentations and 10 unreinforced CS− presentations. After the extinction phase a test phase of 3 unreinforced CS+ presentations and 3 unreinforced CS− presentations was presented. During the whole procedure inter-trial intervals (ITIs) were 20, 25, or 30 s. The order of the length of the ITI was random, with the restriction of only two successive lengths. For participants in the AAA condition, extinction occurred in Context A, while for participants in the ABA condition extinction took place in a different context (Context B). The basic design for testing conditioning effects was a 2 (CS+ vs. CS−) × 10 (trial) within subjects design. Similarly, the basic design for testing renewal effects was a 2 (CS+ vs. CS−) × 3 (trial) within subjects design.

### Materials, Apparatus, and Recording

#### Stimulus Materials

(see Text S1) Two neutral pictures served as CS+ and CS−. Each picture portrayed a black and white cartoon-like drawing of the head of a person. During intervals between the pictures, a white screen was presented. The CS+ and CS− were presented for 9 s each.

#### Genital vibrostimulation

(US) was provided only during the acquisition phase, 8 s following the start of each CS+ for 2 s. For male participants, the vibrotactile genital stimulation was administered by means of a hands-off ring-shaped vibrator (Aquasilks), which was placed by the participants themselves just below the coronal ridge. For women, a small hands-off vibrator (2 cm diameter) was used, placed on the clitoris. The participants were instructed to place the vibrator in such a way it was *most sexually stimulating*.

#### Context manipulation

Contexts were manipulated by illuminating the experimental room in either a pink or a yellow light. Lighting was supplied by a frame with six fluorescent tubes of 36 W (two pink and four yellow tubes), resulting in a slightly dimmed colored illumination of the whole room. The experimenter controlled the lighting from an adjacent room.

#### Male genital sexual arousal

was measured using an indium/gallium-in-rubber penile gauge assessing changes in circumference of the penis [37]. The penile gauges were calibrated before each laboratory session using a set of calibrated rings [38]. Participants were instructed to place the gauge midway along the penile shaft. Changes in electrical output caused by expansion of the gauge were recorded by a continuous DC signal. The indium/gallium penile gauges were disinfected after each use, according to Sekusept plus disinfection procedure. Sekusept plus contains Glucoprotamine, which action spectrum covers bacteria including mycobacteria, fungi and viruses (e.g. Human Papillomavirus [HPV]) (MedCaT B.V.).

#### Women's genital arousal

was measured using a vaginal photoplethysmograph assessing vaginal pulse amplitude (VPA) [39] (see *Text S1*). The photoplethysmograph is a menstrual tampon-sized device containing an orange-red light source and a photocell. The light source illuminates the capillary bed of the vaginal wall and the blood circulation within it. The photoplethysmograph was disinfected at the LUMC by means of a plasma sterilization procedure between uses. Plasma sterilization is a highly effective method for the complete removal of all organic (and certain in-organic) material. Genital response was measured continuously during resting baseline, preconditioning, acquisition, extinction, and test phases.

#### Subjective Ratings

Ratings of affective value, sexual arousal and US expectancy were collected during the preconditioning-, extinction- and test phase. Participants were first asked to rate, after each CS presentation, the affective value of the CSs by answering the question “*What kind of feeling does this picture evoke in you*?” on a seven-point Likert scale on a keyboard that varied from *very negative* to *very positive*. Then, subjective sexual arousal was rated by answering the question “*How sexually arousing is this picture to you*?” on a seven-point scale that varied from *not sexually arousing at all* to *very sexually arousing*. Then, US expectancy was rated by answering the question “*To what extent did you expect a vibration after this picture*”? on a seven-point scale labeled from ‘*certainly no vibration’* to ‘*certainly a vibration’*.

### Other Measures

#### Approach Avoidance Task

(AAT; see *Text S1*) [40]. This task assesses approach and avoidance motivational processes by requiring participants to respond to irrelevant feature of pictures by either pulling a joystick handle toward them or by pushing it away. The amount of time required to execute these actions is the dependent variable. Participants were presented with the CS+ and CS− pictures from the experiment, as well as neutral pictorial objects and cartoon faces resembling the CSs. Literature supports the AAT's validity in measuring approach/avoidance motivational processes [41].

### Procedure

Each participant was tested individually by a trained experimenter of the same sex. After participants had given informed consent, the experimenter explained the experimental procedure and the use of the plethysmograph, penile gauge and vibrator. Then, the experimenter left the room to allow the participant to place the genital devices privately. Further instructions were given through written instructions on the monitor. Then a 5-minute resting period followed, during which a neutral film was played and baseline measurements of genital response were collected during the last 2 minutes. Then the preconditioning, acquisition, extinction and test phases followed.

Immediately after the experimental procedure and after the participant removed the genital devices, the AAT was presented in the experimental room with the same lighting conditions as in the original acquisition context (A). After completing the AAT, participants filled in questionnaires about demographics, and sexual function (e.g., FSFI, IIEF). Finally, an exit interview questionnaire was administered.

### Data Reduction, Scoring and Analysis

A software program (VSRRP98; developed by the Technical Support Department of Psychology, University of Amsterdam) was used to analyze the genital data. Mean penile circumference or mean VPA level during the 2-minute resting baseline period was calculated. Genital responses to the CSs were scored in three latency windows: during 4–8, 9–12 and 13–16 s following CS onset, respectively FIR (first interval response), SIR (second interval response) and TIR (third interval response). For FIR, SIR and TIR, change scores were calculated for each CS presentation by subtracting mean genital resting baseline from genital measures following CS presentation.

For genital responses, effects were tested separately for men and women, with Mixed ANOVA's (General Linear Model in SPSS), with Stimulus and Trial as within-subject factors and Condition as between subjects factor.

Analyses of subjective measurements and AAT scores were conducted for men and women combined. For subjective ratings, effects were tested with Mixed ANOVA, with Stimulus and Trial as within-subject factors and Condition and Gender as between saubjects factor.

The Greenhouse–Geisser correction was applied to adjust for violation of the sphericity assumption in testing repeated measures effects. Preconditioning, acquisition, extinction, and test phases were analyzed separately. Effect sizes are reported as proportion of partial variance (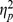) [42].

For the AAT, bias scores were calculated by subtracting median approach RT from median avoid RT for each image category. Median RTs were used because they are less sensitive to outliers than means. A positive bias score indicated a relatively faster approach compared to avoid RTs, whereas a negative score indicated a relatively faster avoid compared to approach RTs. To compare the AAA and ABA condition, bias scores were analyzed using ANOVA.

## Results

Of the 62 women tested, genital data of 2 participants were left out. One data point in the acquisition phase of a male participant was discarded as outlier because this measure was above 4 SD from the mean (inclusion of this data point did not change results). Results from the AAT are based on 99 participants (see *Text S1* for specific information).

### Subject characteristics

Participants were randomly assigned to one of the two context conditions with the restriction that conditions were matched on sex as close as possible: AAA (N =  49; Men, *n*  =  20) and ABA (N =  53; Men, *n*  =  20). Men and women did not differ in age, in sexual functioning, nor in prior experience with vibrostimulation across conditions, see Table 1 *Subject characteristics*.

**Table 1:** Subject characteristics.

*Variable:*	Men					Women					Men & Women				
	AAA (n = 20)		ABA (n = 20)			AAA (n = 29)		ABA (n = 33)			Men (N = 40)		Women (N = 62)		
	MEAN	SD	MEAN	SD	*p*	MEAN	SD	MEAN	*SD*	*p*	MEAN	SD	MEAN	SD	*p*
**Age (years)**	22.3	2.6	24.9	6.5	*.11*	21.5	2.8	22.5	2.9	*.60*	23.6	5.0	22	2.8	*.04*
**Sexual Functioning (IIEF/FSFI- score)**	33.5	5.5	35.8	6.2	*.21*	26.6	2.4	26.4	2.9	*.83*					
**Prior Experience Vibrostimulation**	1.8	1	1.7	1	*.75*	3	1.3	2.9	1.2	*.85*	1.7	1	3.0	1.3	*<.01*
**Pleasantness US**	3.4	1.1	3.2	0.7	*.62*	3.4	0.9	3.3	0.8	*.53*	3.3	0.9	3.3	0.8	*.72*
**US Perceived as Sexually Arousing**	3.1	1.1	2.7	0.7	*.19*	3.1	0.9	3.1	0.8	*.41*	2.9	0.9	3.1	0.9	*.18*
**Declared Sexual Arousal**	2.4	0.9	2.1	0.7	*.30*	2.6	0.8	2.5	0.8	*.79*	2.2	0.8	2.6	0.8	*.06*

Descriptive subject variables for men and women, and for each condition.

Notes: Scale Prior experience vibrostimulation: 1 (never) – 5 (very often); Scale Pleasantness US: 1 (not pleasant at all) - 5 (very pleasant); Scale US perceived as sexually arousing: 1 (not sexually arousing at all) – 5 (very sexually arousing); Scale Declared sexual arousal: 1 (not sexually aroused) – 5 (very sexually aroused).

Fourteen women indicated not to have a stable heterosexual relationship at the time of the study, and six women indicated not having had sexual activity with a partner during the last weeks, hence resulting in a low FSFI score.

Because the exit interview revealed men and women differed slightly in the way they had perceived the US, additional analyses were conducted leaving out the five male participants who reported to have experienced the vibrotactile stimulation as unpleasant or not sexually arousing at all. However, all additional analyses revealed no different pattern of responding as reported.

### Genital Sexual Arousal

#### Preconditioning phase

Responses for three latency windows were analyzed: first, second, and third interval response (FIR, SIR, TIR, see*Text S1*). Analyses were conducted to verify equal levels of penile circumference and VPA in response to the CSs during the preconditioning phase. For FIR and SIR, no difference in circumference or VPA following presentation of the CS+ and CS− was found, *p*s > .11. VPA TIR in response to the CS− was higher as compared to the CS+, although this difference did not reach conventional level of significance, VPA, *p* < .08, 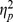 =  .06.

#### Acquisition phase. *Men:*

**Figure 2:**
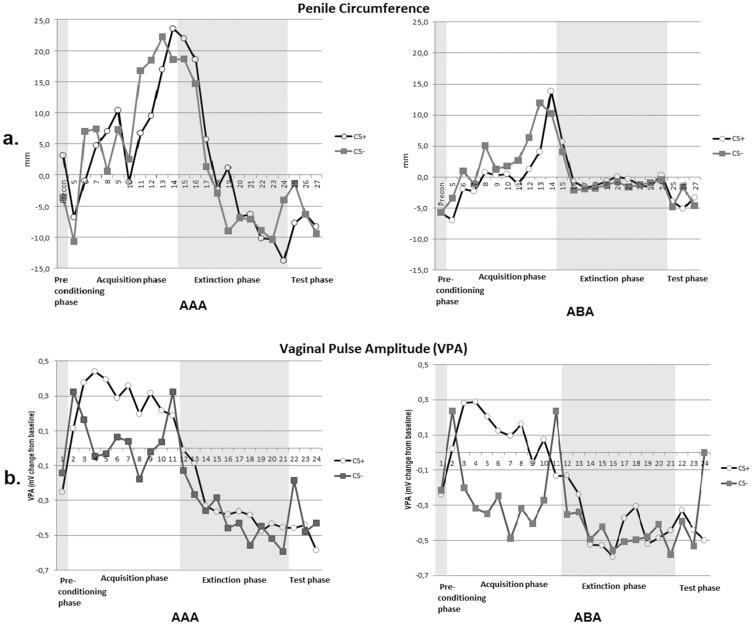
Mean penile circumference change scores (a.) and Mean vaginal pulse amplitude (VPA) change scores (b.) during the third interval response window (TIR) following the CS+ and CS− during the preconditioning phase, acquisition phase, extinction phase and test phase for the two conditions AAA and ABA. Note that during the acquisition phase, the response represents responding to the CS+ plus the US.

Figure 2,a summarizes penile circumference (TIR) to CS+ and CS− across trials for both conditions separately. The analysis of penile circumference in the acquisition phase revealed a main effect of Stimulus, FIR, *F* (1, 36) =  12.39, *p*< .01, 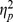 = .26, SIR, *F*(1, 35) =  83.68, *p*<.01, 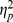 =  .70, TIR, *F*(1,35) =  16.96, *p*< .01, 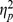 = .33, meaning the vibrostimulation resulted in a genital response, as can be seen in Figure 2,a. Contrary to the expectation, penile circumference to CS− was larger as compared to CS+. No effects for Trial were observed, all *p*s> .24, and no significant 2 (Stimulus) × 10 (Trial) interaction was found, all *p*s> .39. This pattern of acquisition did not differ between conditions as reflected by non-significant 2 (Stimulus) × 10 (Trial) × 2 (Condition) and 2 (Stimulus) × 2 (Condition) interactions, all *p*s>.41.

#### *Women:*

In line with previous studies [43], [44], the analyses of VPA during the acquisition phase did not reveal a main effect of Stimulus on FIR, *p*< .08, and SIR, *p* =  .28, whereas it did on TIR, showing that VPA was higher following the presentation of the CS+ plus vibrostimulation than following the CS−, *F*(1, 56) =  27.74, *p*< .01, 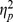 =  .33. As can be seen in Figure 2,b, the vibrostimulation resulted in a genital arousal response. No significant effects for Trial were observed, FIR, *p* =  .53; SIR *p* =  .07; TIR *p* =  .15. However, an interaction effect of Stimulus and Trial for VPA TIR was found, *F*(5, 268)  =  6.73, *p*< .01, 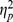 =  .11, indicating differentiation between genital responding to CS+ plus vibrostimulation and CS− over trials. This pattern of acquisition did not differ between conditions as reflected by a non-significant 2 (Stimulus) × 10 (Trial) × 2 (Condition) effect, *p* =  .85.

#### Extinction phase. *Men*:

The 2 (Stimulus) × 10 (Trial) × 2 (Condition) Mixed ANOVA revealed no overall larger penile responses to CS+ than to CS-, and showed no significant Stimulus X Trial interaction, *p*s> .17. This indicates that there was no difference in penile responding towards the CS+ and CS-, and this pattern of responding did not change across extinction trials. In addition, no differences between the conditions were seen, *p*s> .30. An additional 2 (Stimulus) X 2 (Phase; Mean trial 1–4 precon and the first extinction trial) Mixed ANOVA for penile circumference, revealed a trend for Stimulus on FIR, *F*(1, 37) =  2.92, *p*< .10, SIR, *F*(1, 37) =  2.85, *p* =  .10, and TIR, *F*(1, 37) =  2.99, *p* =  .09. However, no interaction effect for Stimulus and Trial was observed, *p*s> .80, indicating no conditioned differential responding on the first extinction trial. Analysis of the entire extinction phase yielded no main effect for Trial, FIR *p* =  .23; SIR *p* =  .23; TIR *p* =  .23. The additional 2 (Stimulus) X 2 (Phase; Mean trial 1–4 precon and the last extinction trial) Mixed ANOVA revealed no main effect for Stimulus, *p*s> .58. In summary, the picture that was reinforced by genital vibrostimulation during the acquisition phase did not elicit greater penile circumference during the extinction phase, and both conditions did not differ in genital responding to the CSs, see Figure 2, a.

#### *Women*:

Because extinction of conditioned responding cannot be expected when there is no acquisition of conditioned responding, VPA FIR results are not reported. As expected a significant main effect for Stimulus was found, SIR, *F*(1, 57) =  4.73, *p*< .03, 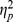 =  .04; TIR, *F*(1, 56) =  5.78, *p* =  .02, 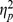 =  .09, meaning the picture that was reinforced by clitoral vibrostimulation during the acquisition phase did elicit higher VPA during the extinction phase, as can be seen in Figure 2, b. Most crucial to our hypothesis, the ANOVA showed no significant interaction effect between Stimulus and Trial, SIR, *p* =  .21, TIR, *p* = .21, meaning no extinction effect. The analysis also revealed that this pattern of differential responding towards CS+ and CS− did not differ between conditions, SIR, *p* =  .30, TIR, *p* =  .91. As expected, additional analysis of the first extinction trial yielded significant differences for VPA SIR *F*(1, 57) =  7.74, *p*< .01, 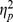 =  .12, and TIR, *F*(1, 58) =  3.96, *p* =  .05, 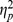 =  .06. Also, the analysis of the last extinction trial yielded significantly higher VPA in response to the CS+ than in response to the CS− for VPA SIR, *F*(1, 57) =  4.31, *p* =  .04, whereas no difference in VPA TIR could be detected, *p* =  .12. Again, the pattern of differential responding towards CS+ and CS− did not differ between conditions, first extinction trial: *p*s> .24, last extinction trial: *p*s> .41. However, there was a main effect of Trial, indicating VPA was decreasing over time, SIR, *F*(4, 228) =  3.66, *p*< .01, 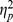 = .06; TIR, *F*(4, 215) =  3.88, *p*< .01, 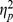 =  .07. In summary, the conditions did not differ in conditioned responding during the extinction phase: AAA and ABA showed an equal differential VPA responding to the picture that was reinforced by clitoral vibrostimulation during the acquisition phase, and for both conditions this differential responding showed no complete extinction across trials. However, for both conditions VPA was decreasing over time (see Figure 2, b).

#### Test phase

Because recovery of conditioned responding cannot be expected when there is no acquisition of conditioned responding, results for men were not reported for the sake of brevity. For the same reason, VPA FIR results were not reported.

#### *Women*:

The analysis of main interest, the 2 (Stimulus) X 2 (Phase; last extinction trial and first test trial) 2 X (Condition) Mixed ANOVA, yielded a trend for Stimulus X Trial X Condition, *F*(1, 56) =  3.10, *p* = . 08, 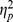 =  .05. The 2 (Stimulus) X 3 (Trial) X 2 (Condition) analysis of the test phase for VPA SIR, yielded borderline significance on VPA SIR, *F*(1, 102) =  3.09, *p*< .06. Inspection of Figure 2, b, suggests these effects may be explained by unexpectedly large responses to the CS−. Therefore, we additionally conducted a separate 2 (Phase) X 2 (Condition) ANOVA for only CS+ responses on the last extinction trial and first test trial, see also [16], [19]. However, no significant interaction effect was seen for Stimulus X Condition, *p* =  .19. For VPA TIR the interaction most crucial to our hypothesis, Stimulus X Phase X Condition, yielded no significance, *p* =  .19. The analysis of VPA TIR on the last extinction trial and first test trial, yielded a main effect for Stimulus, *F*(1, 56) =  4.18, *p*< .05, 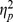 =  .07. Contrary to the expectations, no interaction effect for Stimulus X Phase X Condition was found, *p* =  .24. Additional analysis of only CS+ responses during TIR, yielded no significance, *p* =  .39. Hence, women showed no increased conditioned genital responding to the CS+ upon changing the context after extinction.

### Subjective measures

#### Preconditioning phase

For US expectancy and affective value, no difference in responding to the CSs was found between conditions and between men and women, all *p*s > .20. However, for subjective sexual arousal there were marginally significant interaction effects for Stimulus X Gender, *p*< .09, and Stimulus X Condition X Gender, *p* =  .06, meaning men and women tended to differ in ratings of subjective sexual arousal towards the CSs, with men rating the CS+, and women rating the CS− as slightly more arousing.

#### Extinction phase. *US Expectancy*:

**Figure 3:**
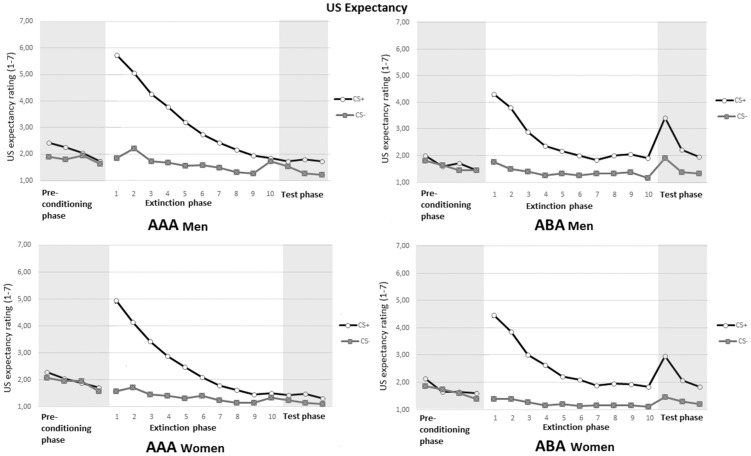
US expectancy ratings following the CS+ and CS− during the preconditioning phase, extinction phase and test phase for men (top) and women (bottom) in the two conditions AAA and ABA.

As can be seen in Figure 3, both conditions showed a robust increase of differential responding towards CS+ vs. CS− after the acquisition phase, and a decrease in this differential responding over trials. Analysis of US expectancy ratings during the preconditioning phase (Mean trial 1–4) and the first extinction trial, revealed a main effect for Stimulus, *F*(1, 97) =  128.07, *p*< .01, 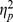 =  .57, and an interaction effect for Stimulus and Trial, *F*(1, 97) =  133.49, *p*< .01, 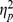 =  .58, indicating a conditioning effect. The 2 (Stimulus) × 10 (Trial) × 2 (Condition) × 2 (Gender) Mixed ANOVA of the extinction phase yielded a significant Stimulus X Trial interaction, *F*(3, 283) =  47.39, *p*< .01, 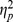 =  .34. No significant Stimulus X Trial X Condition interaction was found, *p* =  .16, meaning both conditions showed an equal loss of expecting the US after presentation of the CS+. Analysis of expectancy ratings on the first extinction trial and the last extinction trial, revealed a main effect for Stimulus, *F*(1, 97) =  135. 09, *p*< .01, 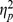 =  .58, and an interaction effect for Stimulus X Trial, *F*(1, 97) =  118.95, *p*< .01, 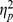 =  .55, indicating extinction of conditioned responding. Also a trend was detected for Stimulus X Trial X Condition, *F*(1, 97) =  2.97, *p*< .09, with the AAA condition showing stronger loss of US expectancy. Analysis of the first extinction trial yielded a significant main effect for Stimulus, *F*(1, 97) =  147.36, *p*< .01, 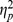 = .60. Likewise, analysis of the last extinction trial also yielded a significant main effect for Stimulus, *F*(1, 95) =  9.61, *p*< .01, 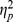 =  .09, but also an interaction effect for Stimulus X Condition, *F*(1, 95) =  4.02, *p*< .05, 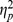 =  .04. This indicates there was still a difference in the ABA condition in US expectancy in response to the CS+ and the CS− on the last extinction trial. In sum, men and women showed an equal loss of expecting the US after presentation of the CS+.

#### *Affective Value*:

**Figure 4:**
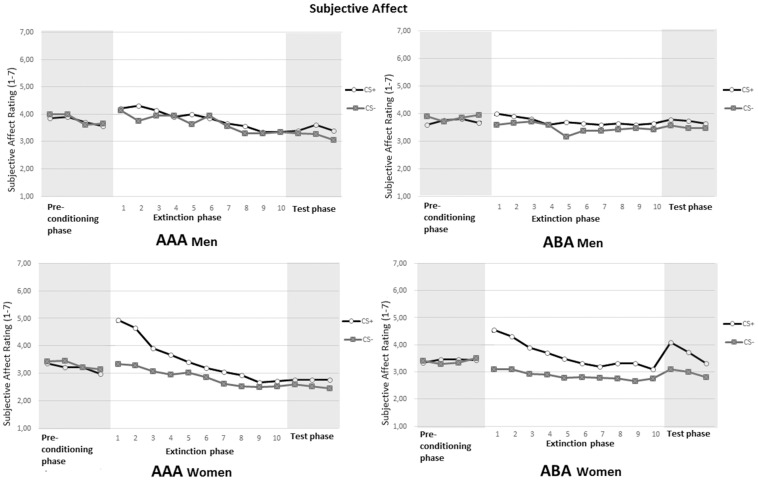
Ratings of subjective sexual arousal following the CS+ and CS− during the preconditioning phase, extinction phase and test phase for men (top) and women (bottom) in the two conditions AAA and ABA.

Men and women differed in conditioned responding after the acquisition phase, see Figure 4. For women, both conditions showed a more robust increase of differential responding towards CS+ vs. CS− after the acquisition phase, and a decrease in this differential responding over trials.

The 2 (Stimulus) X 2 (Phase; Mean Precon trial 1–4 and first extinction trial) X 2 (Condition) X 2 (Gender) Mixed ANOVA of the affective value ratings revealed an interaction effect for Stimulus X Trial, *F*(1, 97) =  29.73, *p*< .01, 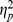 =  .24. Also an interaction effect was found for Stimulus X Phase X Gender, *F*(1, 97) =  16.95, *p*< .01, 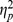 = .15. Analyses of the preconditioning phase and first extinction trial for men and women separately, yielded no significant interaction for Stimulus X Phase for men, *F*(1, 38) =  1.59, *p* =  .22. This indicates there was no conditioned responding on subjective affect for men, as can be seen in Figure 4. For women, this analysis yielded a significant interaction effect for Stimulus X Phase, *F*(1, 59) =  52.92, *p*< .01, 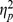 =  .47.

As expected, analysis of the extinction phase showed a significant Stimulus X Trial interaction, *F*(4, 378) =  8.92, *p*< .01, 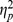 =  .09, indicating that the difference in rated subjective affect between CS+ and CS− gradually decreased across trials, which constitutes extinction. The ANOVA yielded no Stimulus X Trial X Condition interaction *F*(4, 378) =  0.62, *p* =  .65, but did yield a significant Stimulus X Trial X Gender interaction, *F*(4, 378) =  7.52, *p*< .01, 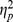 =  .07.

Additional analysis of the first and the last extinction trial, revealed interaction effects for Stimulus and Trial, *F*(1, 96) =  17.66, *p*< .01, 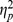 =  .16, and Stimulus X Trial X Gender, *F*(1, 96) =  14.37, *p*< .01, 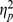 =  .13. No significant interaction effect for Stimulus X Trial X Condition was found, *p* =  .54. Meaning, although both conditions showed equal loss of conditioned responding, this effect can be attributed to women's responding. Additional analyses of the first and the last extinction trial for men and women separately, revealed no interaction effect for Stimulus X Trial for men, *F*(1, 37) =  0.10, *p* =  .76, meaning no extinction occurred.

As expected, this analysis for women revealed a significant interaction effect for Stimulus X Trial, *F*(1, 59) =  34.47, *p*< .01, 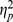 =  .37, indicating extinction. Analysis of the first extinction trial yielded a significant main effect for Stimulus, *F*(1, 97) =  28.19, *p*< .01, 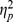 =  .23, and significant interaction effect for Stimulus X Gender, *F*(1, 97) =  19.28, *p*< .01, 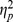 =  .17. Analysis of the last extinction trial still yielded a significant main effect for Stimulus, *F*(1, 97) =  5.69, *p* =  .02, 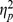 =  .06, indicating differential responding towards the CS+ and CS−.

#### *Subjective Sexual Arousal*:

Figure 4 shows increased ratings of sexual arousal towards the CS+ on the first trials of the extinction phase, which constitutes conditioned responding. The 2 (Stimulus) X 2 (Phase; Mean Precon trial 1–4 and first extinction trial) X 2 (Condition) X 2 (Gender) Mixed ANOVA of ratings of sexual arousal, yielded a significant interaction for Stimulus X Phase X Gender, *F*(1, 94) =  5.69, *p* =  .02, 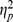 = .06. Subsequent analysis of the preconditioning phase (Mean trial 1–4) and the first extinction trial for men and women separately, yielded significant interactions for Stimulus X Phase for men, *F*(1, 36) =  6.73, *p*< .02, 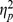 =  .16, and women, *F*(1, 58) =  38.20, *p*< .01, 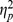 =  .40, indicating conditioned responding. However, as can be seen in Figure 4, women displayed a stronger conditioned responding. Moreover, in line with the expectation, the analysis of the extinction phase yielded a significant Stimulus X Trial interaction, *F*(4, 404) =  6.93, *p*< .01, 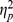 =  .07, meaning a decrease of conditioned responding over trials. No Stimulus X Trial X Condition interaction was found, *p* =  .96, but again a significant interaction for Stimulus X Trial X Gender, *F*(4, 404) =  3.72, *p*< .01, 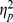 =  .04. For subjective sexual arousal, both conditions did not differ in loss of differential responding, that is extinction. However, women showed a greater loss of differential ratings to CS+ and CS− during the extinction phase than men, as can be seen in Figure 4.

Analysis of the first and last extinction trial yielded a significant interaction effect for Stimulus X Trial, *F*(1, 97) =  21.0, *p*< .01, 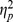 = .18, indicating extinction of conditioned subjective sexual arousal. No significant interaction effect was found for Stimulus X Trial X Condition, *p* =  .93, indicating no differences between the conditions in extinction of conditioned responding. Again an interaction effect was found for Stimulus X Trial X Gender, *F*(1, 97) =  7.32, *p*< .01, 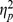 = .07. Separate analyses for men and women for the first and the last extinction trial were conducted. For men, this analysis yielded no significant interaction effect for Stimulus and Trial, *p* =  .27, and Stimulus X Trial X Condition interaction, *p* =  .80, meaning no extinction, with no differences between conditions. For women, this analysis yielded significance for Stimulus X Trial, *F*(1, 60) =  37.22, *p*< .01, 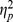 = .38, meaning extinction of conditioned differential responding. No differences between groups were observed in this loss of conditioned responding, Stimulus X Trial X Condition interaction, *p* =  .84.

Analysis of the first extinction trial yielded a significant main effect for Stimulus, *F*(1, 97) =  41.38, *p*< .01, 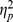 =  .30, and an interaction effect for Stimulus X Gender, *F*(1, 97) =  4.36, *p* =  .04, 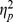 =  .04. Analysis of the last extinction trial also revealed a main effect for Stimulus, *F*(1, 97) =  9.67, *p*< .01, 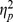 =  .09, indicating there still was differential responding towards the CS+ and CS− on the last extinction trial, and men and women did no longer differ therein.

#### Test phase. *US Expectancy*:

The 2 (Stimulus) X 2 (Phase; last extinction trial and first test trial) X 2 (Condition) X 2 (Gender) Mixed ANOVA of ratings of US expectancy, yielded significance for Stimulus X Phase, *F*(1, 94) =  10.01, *p*< .01, 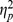 = .10, and for the interaction of main interest, Stimulus X Phase X Condition, *F*(1, 94) =  8.44, *p*< .01, 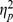 = .08. Subsequent analysis of the test phase yielded a significant interaction for Stimulus X Trial, *F*(2, 153) =  9.11, *p*< .01, 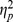 = .09, and for Stimulus X Trial X Condition, *F*(2, 153) =  8.31, *p*< .01, 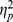 = .08. As can be seen in Figure 3, men and women showed recovery of US expectancy towards the CS+ on the test trials, as result of context switch. Inspection of Figure 3 also suggests increased responding towards the CS− for men and women in the ABA condition. Additional analysis of the last extinction trial and first test trial for only CS− responses, yielded a significant interaction effect for men for Stimulus X Condition, *F*(1, 94) =  12.05, *p*< .01, 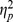 = .11, indicating increased US expectancy would also follow the CS− as a result of context switch.

#### *Affective Value*:

The analysis of the last extinction trial and first test trial, yielded significant interactions for Stimulus X Condition, *F*(1, 95) =  5.32, *p* =  .02, 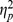 = .05, and most crucial to our hypothesis for Stimulus X Phase X Condition, *F*(1, 95) =  5.76, *p* =  .02, 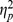 = .06. Moreover, significant interactions were also found for Stimulus X Condition X Gender, *F*(1, 95) =  4.21, *p* =  .04, 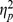 = .04, and Stimulus X Phase X Condition X Gender, *F*(1, 95) =  8.20, *p*< .01, 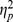 = .08. Since men did not show conditioned responding after the acquisition phase on affective value ratings, further results for men were not reported.

Separate analyses for women, revealed a significant interaction effect, *F*(1, 59) =  13.82, *p*< .01, 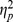 = .19. Inspection of Figure 4 suggests also increased responding towards the CS− on the first test trial for women in the ABA condition. Additional analysis of the last extinction trial and the first test trial for only affective value ratings towards CS− yielded a trend, *F*(1, 59) =  3.01, *p*< .09. Meaning affective value towards the CS− also increased as a result of context switch after extinction.

Analysis of the test phase, yielded trends for Stimulus X Condition, *p*< .08, and for Stimulus X Trial X Condition, *p* =  .07. Furthermore, the analysis yielded a trend for Stimulus X Condition X Gender, *p*<.06. The interaction effect of Stimulus X Condition indicates that the conditions differed in differential responding to the CS+ and CS−. The ABA condition showed recovery of conditioned responding and rated the CS+ as more positive as compared to the CS−. The significant interaction effect of Trial X Condition X Gender, indicates that there was a difference in responding between the two conditions between men and women, with only women showing recovery of conditioned responding, as can be seen in Figure 4. Additional analyses of the renewal phase for women, yielded significant interactions for Stimulus X Trial X Condition, *F*(2, 101) =  3.41, *p* =  .04, 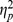 = .06.

#### *Subjective Sexual Arousal*:

**Figure 5:**
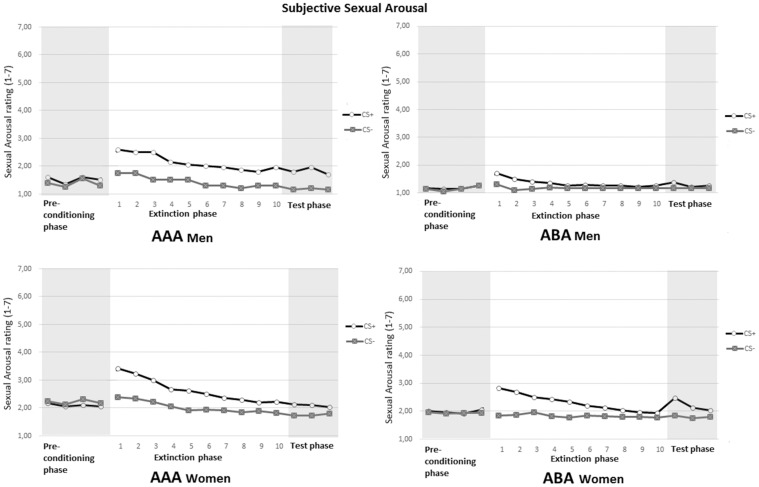
Subjective affect ratings following the CS+ and CS− during the preconditioning phase, extinction phase and test phase for men (top) and women (bottom) in the two conditions AAA and ABA.

Analysis of the last extinction trial and first test trial, yielded significance for Stimulus X Condition, *F*(1, 94) =  8.21, *p*< .01, 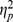 = .08, and most important, for Stimulus X Phase X Condition, *F*(1, 94) =  5.17, *p* =  .03, 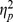 = .05. Also a significant interaction effect for Stimulus X Condition X Gender was seen, *F*(1, 94) =  5.41, *p* =  .02, 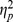 = .05. Separate analyses for men and women, revealed no interaction effect for Stimulus X Phase X Condition in men, *p* =  .54, whereas this analysis yielded a significant effect in women, *F*(1, 58) =  7.35 *p*< .01, 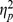 = .11, meaning increased conditioned responding after context switch was observed only in women. Subsequent analysis of the test phase revealed significant interaction effects for Stimulus X Trial, *F*(2, 175) =  7.64, *p*< .01, 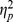 = .08, and for Stimulus X Condition X Gender, *F*(1, 93) =  4.63, *p* =  .03, 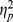 = .05. Inspection of Figure 5 suggests these effects may be explained by larger responses by women to CS+ on the first test trial for the ABA condition, as compared to men. For men, additional analysis of the test phase, yielded no significant interaction for Stimulus X Trial, *p* =  .25, whereas for women this interaction was significant, *F*(2, 101) =  9.39, *p*< .01, 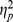 = .14. For men only a main effect for Stimulus was found, *F*(1, 35) =  4.69, *p*< .04, 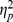 = .12.

### Approach Avoidance Tendencies

*t*-tests were used to test if bias scores deviated significantly from zero within each condition, see Table 2. Differences in AAT bias scores were analyzed with mixed ANOVA with Gender and Condition as between-subject factor and Image as within-subject factor (CS+, CS−, CS−alike and neutral objects). Contrary to the expectations, no interaction effect was found for Image X Condition, *p* = .28. Participants from the two conditions did not differ in approach and avoidance tendencies across all stimuli. However, a main effect for Condition was found, *F*(1, 95) =  5.17, *p*< .03, 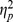 =  .05, reflecting more approach biases towards stimuli for participants in the ABA condition. Contrary to the expectations, there was no main effect for Image, *p* =  .62, but a there was a trend for Image X Gender, *F*(3, 258) =  2.39, *p*< .08, meaning men and women differed in their bias scores. Further testing revealed that men and women differed in CS+ bias score, *t*(97) =  −2.20, *p* =  .03. Women were faster in approaching the CS+ as compared to men. They were however also faster in approaching the CS− although this did not reach conventional level of significance, *t*(97) =  −1.66, *p*< .10.

**Table 2:** One-sample t-test results for Mean Approach Avoidance Task (AAT) bias score for CS+, CS−, CS-alike and neutral images in men and women in the AAA and ABA condition.

		Bias Score	MEAN	SD	*p*
**Men**	**AAA**	**CS+**	17.8	44.8	.*10*
		**CS−**	15.6	60.3	.*27*
		**CS-alike**	26.2	34.6	*< .01*
		**Neutral**	6.3	39.9	.*50*
	**ABA**	**CS+**	21.0	48.1	.*07*
		**CS−**	21.4	46.3	.*05*
		**CS-alike**	23.9	47.6	.*04*
		**Neutral**	44.3	60.1	*< .01*
**Women**	**AAA**	**CS+**	30.2	53.7	*< .01*
		**CS−**	26.6	55.4	*< .02*
		**CS-alike**	8.6	56.4	.*40*
		**Neutral**	10.4	48.4	.*26*
	**ABA**	**CS+**	54.7	56.8	*< .01*
		**CS−**	47.2	57.7	*< .01*
		**CS-alike**	37.4	52.4	*< .01*
		**Neutral**	37.6	61.1	*< .01*

A positive score indicates faster reaction times on approach (pull) trials compared to avoid (push) trials.

### Correlations between Conditioned Responses

To investigate relationships between conditioned responses additional correlational analyses were conducted. We expected that the strength of the conditioned genital response would be positively related to the amount of change in subjective affect and subjective arousal and US expectancy. In addition, it was expected that the strength of the conditioned genital response would be positively related to the CS+ bias score. To investigate these relationships, for genital responses on SIR and TIR and ratings of affect, and subjective sexual arousal and US expectancy, the difference between the response to the CS+ and the CS− during the first trial in the extinction phase was calculated by subtracting the response to the CS− from the response to the CS+. Pearson product-moment correlations between genital difference score during the first extinction trial, affect difference score, subjective sexual arousal difference score, US expectancy ratings difference scores, were calculated (see Table 3).

**Table 3:** Correlations between conditioned genital response, conditioned affective change, conditioned subjective sexual arousal, conditioned US expectancy and conditioned approach and avoidance tendencies towards the CSs for men and women.

		Affective Value	Subjective Sexual Arousal	US Expectancy	Conditioned Genital Response SIR	Conditioned Genital Response TIR	Bias Score CS+	Bias Score CS−
**Men**	**Affective Value**		.001	.187	.517	.326	.315	.160
	**Subjective Sexual Arousal**	.001		.078	.348	.411	.203	.634
	**US Expectancy**	.187	.078		.375	.559	.449	.034
	**Conditioned Genital Response SIR**	.517	.348	.375		< .001	.408	.471
	**Conditioned Genital Response TIR**	.326	.411	.559	< .001		.398	.498
	**Bias Score CS+**	.315	.203	.449	.408	.398		<.001
**Women**	**Affective Value**		.329	.008	.261	.392	.765	.706
	**Subjective Sexual Arousal**	.329		.001	.034	.283	.177	.212
	**US Expectancy**	.008	.001		.051	.037	.589	.864
	**Conditioned Genital Response SIR**	.261	.034	.051		<.001	.043	.113
	**Conditioned Genital Response TIR**	.392	.283	.037	<.001		.029	.339
	**Bias Score CS+**	.765	.177	.589	.043	.029		<.001

Table 3 shows that there were no significant correlations between the strength of the conditioned genital response and conditioned subjective and behavioural measures in men. However, in women, the strength of the conditioned genital response was correlated to the amount of change in subjective arousal and US expectancy. In addition, the strength of the conditioned genital response was also correlated to the magnitude of the CS+ bias score. Interestingly, the CS− bias score did not show such correlations.

## Discussion

The present study contributes to the growing literature on learning mechanisms in sexual behaviors, and provides support of the central feature of Bouton's theory of context dependency of extinction and renewal of conditioned responding in humans. We found evidence for this theory that an extinction procedure indeed does not erase conditioned sexual associations in humans but instead involves new learning that is context dependent. Changing context after an extinction procedure resulted in a significant increase of subjective affect and subjective sexual arousal in women and increased US expectancy ratings to CS+ as compared to CS− in both men and women (ABA condition), whereas no such recovery was observed in the absence of a context change (AAA condition). These results are important, because so far, context dependency of extinction in the sexual domain has not been studied in human studies.

However, it is crucial to mention that not all hypotheses were confirmed. First, no evidence for renewal was found for genital measures in men and women. For men, this can be explained by the fact that genital conditioning effects were not obtained. We will set out possible causes thereof hereafter. To be able to test for renewal, acquisition of conditioned responding has to be ascertained during the acquisition phase. Similarly, this also explains the finding that men did not show renewal of conditioned subjective affect during the test phase.

However, although women showed conditioned genital responding, no renewal of such responding could be observed. For women the absence of renewed genital conditioned responding can be explained by the fact that this is complicated when extinction of such responding is not completely ascertained during the extinction phase. Since women showed no complete extinction of differential genital responding it is not entirely surprising no renewal was observed. In a similar manner, as men did not demonstrate extinction of conditioned subjective sexual arousal, renewal of conditioned responding was made harder to detect during the test phase.

As mentioned before, men did not show conditioned subjective affect. It can be speculated that the difference in US-evaluation between men and women can account for this. It appears that the vibrostimulation was a more effective sexual stimulus for women than for men, resulting in the absence of conditioned male genital response. Rowland and Slob [45] demonstrated that penile vibrotactile stimulation significantly augments erectile response in the presence of an erotic videotape in healthy, sexually functional men. However, they found vibrotactile stimulation alone to produce the lowest level of genital and subjective sexual arousal compared to erotic film. In the present study, men declared to have liked the vibrostimulation as much as women did. Making it not entirely plausible for the vibrostimulation to have less sexual arousing properties for men, also reflected by clear conditioning effects on subjective measures of sexual arousal. Nevertheless, future studies on male sexual learning may consider vibrotactile stimulation combined with erotic film clips as US. In addition, it is suggested women have more erotic ‘plasticity’ [35], and men are more responsive to explicit erotic visual stimuli [33]. Results from the present study and another study from our lab (Brom et al., in preparation) support this notion. Using the same paradigm, but with sexually relevant CSs as the only difference, robust conditioned genital and subjective sexual arousal and affect was observed also in men, while making use of the same US. Therefore it seems that combination of a non-visual sexual US and neutral CSs is not sufficient to elicit conditioned genital responding in men. With respect to genital arousal, the present study contributes to the accumulating evidence [43], [44] that women can be sexually conditioned to initially neutral stimuli, whereas our results do not support such a straightforward mechanism in men, at least, when making use of a tactile US. However, making use of sexually explicit visual stimuli as US, conditioned responses towards an initial neutral CS (a penny jar) were observed by Plaud and Martini [46]. It could be that once sexual preferences are established [32], men are less susceptible to sexual learning to cues that differ too much from their developed preference [23], [34], [47]. However, future research in men and women, making use of both neutral and sexual relevant CSs and visual and vibrotactile USs should be done to be conclusive about this.

Although the finding that men did not show conditioned genital response is in line with earlier sexual conditioning studies [24], these findings oppose the existing idea that men are more receptive to sexual conditioning than women [9], [23]. More studies on sexual learning in both sexes are needed before we can draw any firm conclusions about gender differences in sexual conditionability. The observed differences between men and women may not reflect pure gender differences in sexual conditionability, but may also be explained by differences in sample size and US effectiveness. In addition, we also should mention that sexually conditioned responses have generally been found to be small, especially with a neutral CS [24], [48]. For example, in their sexual conditioning experiment, Klucken et al. [25] did also not find CRs (n = 40), but making use of an increased number of participants (n = 100) Klucken et al. [26] did. Therefore, an explanation for the missing results could be decreased power.

This study is the first investigating whether initially neutral cues will elicit approach tendencies through their mere pairing with a sexually rewarding outcome. Contemporary emotion theories propose that sexual arousal, like any emotion, is a composite of subjective experience, physiological activity, and action disposition [49]–[51]. Some theorists state emotions are primarily action tendencies that are reflected in physiological activity and subjective response [52], [53]. In such a framework, the fact that a CS elicits sexual arousal response after pairing with a sexually rewarding US implies that the CS also elicits an approach tendency: the approach tendency installed through Pavlovian reward learning is translated into overt action. Although women in the AAA condition had an approach bias towards the CS+ and CS−, and ABA condition towards all stimuli, in the present study, men and women differed in implicit approach tendencies towards the stimulus that was paired with vibrostimulation, with women significantly faster approaching the CS+ than men. In women the CS+ elicited a more robust sexual arousal response as compared to men. This conditioned female sexual response translated into subjective experience, physiological measures and in action disposition. Given the finding that a less robust conditioned male sexual response was observed, strong approach tendencies could not be expected.

Contrary to expectations, but in line with results from another conditioning study from our lab (Brom et al. in preparation), men showed a smaller penile circumference in response to the CS+ compared to the CS− during the acquisition on the timeframes during vibrostimulation and also on timeframes when vibrostimulation no longer was applied. This finding does not lend itself to unambiguous interpretation. However, former research on automatic processing of sexual stimuli also found male genital responses to be opposite to the predictions: genital responses towards sexually primed targets were lower than responses to neutrally primed targets [50]. Those results were explained by physiological processes of penile erection. During the initial phases of erectile response, the penis undergoes an increase in length, and this is associated with a simultaneous decrease in circumference. Therefore, the physiology of penile erection may also account for the results found in the present study.

Quite puzzling is the observation of significant renewal effects for the CS− were observed on different measures. Vervliet, Baeyens, Van den Bergh and Hermans [54] noted that this increase in responding is quite common in studies on human spontaneous recovery and reinstatement. They suggested that this increased responding to the CS− can be explained by the CS− no longer being a neutral control stimulus in the test phase. It is possible that in the acquisition phase the CS− acquires inhibitory associations with the US. As a consequence of context change, this inhibition may be disrupted. According to Vervliet and colleagues the CS− may therefore not be the best control stimulus, as it may share the basic process of extinction: inhibition.

A limitation of the present study is the absence of a between subjects (unpaired) control group. Without such a control group it is difficult to determine whether and what learning has occurred, especially for men. At present it is unclear if the increased genital arousal towards the CS+ and CS− was due to conditioning or to pseudo conditioning. The possibility of sensitization of sexual arousal would translate into increased genital responses across trails, and not in differential responding towards the CS+ and CS− per se [55],[56]. Therefore, making use of such a control group in future research is desirable.

In line with earlier research on conditioning of appetitive responses [20], we demonstrated that not all behavioral and emotional changes produced by classical conditioning are organized in the same fashion. One interesting possibility is that US expectancy and subjective ratings of the CSs are not as much influenced by nonspecific sensitization effects of the US. As expected, results from the present study demonstrated that participants can learn to expect to receive a sexual reward when presented the CS+ and not to receive sexual reward when presented the CS−. Our data suggest that conditioned subjective affect and arousal, and conditioned approach tendencies and genital arousal differ from conditioned US expectancies. This divergence may reflect a more fundamental difference, which raises the question of whether there is evidence for similar discrepancies between such measures in other appetitive paradigms (e.g. nicotine addiction). However, our data did demonstrate that in women conditioned US expectancy is correlated with conditioned affective value, conditioned subjective sexual arousal and conditioned genital arousal. In men, conditioned US expectancy was slightly correlated with conditioned subjective sexual arousal. Interestingly, in men, conditioned subjective sexual arousal is highly correlated with conditioned affective value, whereas in women it is not. This suggests that different response systems do not always behave in synchrony with each other in a sexual conditioning procedure: US expectancy, subjective sexual arousal and subjective affect may go hand in hand during this process of conditioning in men, whereas in women subjective sexual arousal does not seem to increase affective value, or vice versa. Further research should illuminate if this pattern is specific for sexual paradigms or if those behavioral and emotional changes produced by classical conditioning can be found in other appetitive conditioning procedures (e.g. substance addiction).

The present results may have implications for the treatment of sexual disorders with a learned component, like hypo- and hypersexuality. Extrapolating to clinical practice, the renewal of conditioned sexual responding may be observed in the relapse patients experience when leaving treatment context. Supported by results from the present study, it can be concluded that in the treatment of sexual disorders with a learned component it is important to reduce relapse after exposure treatment by generalization of extinction to other contexts and with multiple sexual stimuli. With respect to hypersexuality or paraphilia, this could mean applying treatment techniques in the context (e.g. a red-light district) in which the problematic behavior is experienced.

However, because it is evidently impossible to cover all sorts of situations or stimuli in therapy sessions, there will always be a certain risk for patients to relapse when confronted with a particular object, situation or mental state. Therefore, it may be a highly promising perspective to focus on processes that modulate hippocampus-dependent contextual processing during extinction procedures. The glutamatergic N-methyl-D-aspartate (NMDA) receptor is considered essential for long-term potentiation, a process that underlies learning and extinction [57]. D-cycloserine (DCS), a partial NMDA receptor agonist, has been shown to facilitate extinction of learned fear in rats [58], [59], and in humans to facilitate extinction of fear and addictive behavior [13]. The promising results from the studies on pharmacological agents in aversive extinction memory need to be replicated in appetitive conditioning paradigms, in order to know whether they are also applicable in extinction procedures of appetitive disorders.

In conclusion, this is the first observation of the renewal phenomenon of conditioned sexual responses and sexual reward expectancy in humans. The present research has demonstrated that genital and subjective sexual arousal seem to behave differently with regard to extinction and sensitivity to context changes. The results make clear that sexual arousal or the expectation of sexual reward can come under stimulus control by contextual cues associated with states of sexual reward. This makes clear that basic learning processes play a significant role in the development of human sexual behavior, and emphasizes the importance of future studies on sexual conditioning and related phenomena, and pharmacological influences thereof.

## Supporting Information

Text S1 Supplemental Material.
(DOCX)

Data S1 Genital Data: men.
(SAV)

Data S2 Genital Data: women.
(SAV)

Data S3 Subjective Measures.
(SAV)

## References

[1] Bouton ME (2002) Context, ambiguity, and unlearning: Sources of relapse after behavioral extinction. Biol Psychiatry 52: 976–986. 12437938 10.1016/s0006-3223(02)01546-9

[2] Thewissen R, Snijders SJBD, Havermans RC, van den Hout M, Jansen A (2006) Renewal of cue-elicited urge to smoke: Implications for cue exposure treatment. Beh Res Ther 44: 1441–1449. 10.1016/j.brat.2005.10.010 16375853

[3] Bouton ME, Moody EW (2004) Memory processes in classical conditioning. Neurosci Biobehav Rev 28: 663–674. 15555676 10.1016/j.neubiorev.2004.09.001

[4] Nakajima S, Tanaka S, Urushihara K, Imada H (2000) Renewal of extinguished lever press responses upon return to the training context. Learn Motiv 31: 416–431.

[5] Crombag HS, Grimm JW, Shaham Y (2002) Effect of dopamine receptor antagonists on renewal of cocaine seeking by reexposure to drug-associated contextual cues. Neuropsychopharmacol 27: 1006–1015. 10.1016/S0893-133X(02)00356-1 12464457

[6] Bindra D (1974) A motivational view of learning, performance, and behavior modification. Psychol Rev 81: 199–213. 4424766 10.1037/h0036330

[7] Singer B, Toates FM (1987) Sexual motivation. J Sex Res 23: 481–501.

[8] Di Chiara G (1995) The role of dopamine in drug abuse viewed from the perspective of its role in motivation. Drug Alcohol Depen 38: 95–137. 10.1016/0376-8716(95)01118-i 7671769

[9] Brom M, Both S, Laan E, Everaerd W, Spinhoven P (2014) The role of conditioning, learning and dopamine in sexual behavior: A narrative review of animal and human studies. Neurosci Biobehav Rev 38: 38–59. 24211372 10.1016/j.neubiorev.2013.10.014

[10] Pavlov I (1927) Conditioned Reflexes. Oxford, UKOxford Univ. Press

[11] Delamater AR (2004) Experimental extinction in Pavlovian conditioning: behavioural and neuroscience perspectives. Q J Exp Psychol: Comp Phys Psychol 57: 97–132. 10.1080/02724990344000097 15204112

[12] Hermans D, Craske MG, Susan Mineka S, Lovibond PF (2006) Extinction in Human Fear Conditioning. Biol Psychiatry 60: 361–368. 16503330 10.1016/j.biopsych.2005.10.006

[13] Myers KM, Carlezon WA, Davis M (2011) Glutamate receptors in extinction and extinction-based therapies for psychiatric illness. Neuropsychopharmacol 36: 274–293. 10.1038/npp.2010.88 PMC2994960 20631689

[14] Rescorla RA (2001) Retraining of extinguished Pavlovian stimuli. J Exp Psychol: Anim Behav Pro 27: 115–124. 11296487

[15] Bouton ME (2004) Context and behavioral processes in extinction. Learn Mem 11: 485–494. 15466298 10.1101/lm.78804

[16] Effting M, Kindt M (2007) Contextual control of human fear associations in a renewal paradigm. Beh Res Ther 45: 2002–2018. 10.1016/j.brat.2007.02.011 17451643

[17] Kalisch R, Korenfeld E, Stephan KE, Weiskopf N, Seymour B, et al (2006) Context-dependent human extinction memory is mediated by a ventromedial prefrontal and hippocampal network. J Neurosci 26: 9503–9511. 16971534 10.1523/JNEUROSCI.2021-06.2006 PMC2634865

[18] Stasiewicz PR, Brandon TH, Bradizza CM (2007) Effects of extinction context and retrieval cues on renewal of alcohol cue reactivity among alcohol dependent outpatients. Psychol Addict Behav21: 244–248. 10.1037/0893-164X.21.2.244 PMC3832991 17563145

[19] Vansteenwegen D, Hermans D, Vervliet B, Francken G, Beckers T, et al (2005) Return of fear in a human differential conditioning paradigm caused by a return to the original acquisition context. Beh Res Ther 43: 323–336. 10.1016/j.brat.2004.01.001 15680929

[20] Van Gucht D, Vansteenwegen D, Beckers T, Van den Bergh O (2008) Return of experimentally induced chocolate craving after extinction in a different context: Divergence between craving for and expecting to eat chocolate. Beh Res Ther 46: 375–391. 10.1016/j.brat.2008.01.003 18295187

[21] Kafka M (1994) Paraphilia-Related Disorders–Common, Neglected, and Misunderstood. Harvard rev psychiatry 2: 39–40. 10.3109/10673229409017112 9384878

[22] Kuzma JM, Black DW (2008) Epidemiology, prevalence, and natural history of compulsive sexual behaviour. Psychiatric Clinics of North America, 31. Philadelphia, PA: W. B. Saunders Company. 10.1016/j.psc.2008.06.005 18996301

[23] Pfaus JG, Kippin TE, Centeno S (2001) Conditioning and sexual behavior: a review. Horm Behav 40: 291–321. 11534994 10.1006/hbeh.2001.1686

[24] Hoffmann H, Janssen E, Turner SL (2004) Classical conditioning of sexual arousal in women and men: effects of varying awareness and biological relevance of the conditioned stimulus. Arch Sex Behav 33: 43–53. 14739689 10.1023/B:ASEB.0000007461.59019.d3

[25] Klucken T, Schweckendiek J, Christian J, Merz CJ, Tabbert K, et al (2009) Neural activations of the acquisition of conditioned sexual arousal: effects of contingency awareness and sex. J Sex Med 6: 3071–3085. 19656273 10.1111/j.1743-6109.2009.01405.x

[26] Klucken T, Wehrum S, Schweckendiek J, Merz C J, Hennig J, et al (2013) The 5-HTTLPR Polymorphism is associated with altered hemodynamic responses during appetitive conditioning. Hum Brain Mapp 34: 2549–2560. 22505321 10.1002/hbm.22085 PMC6870347

[27] Berridge KC (1996) Food reward: brain substrates of wanting and liking. Neurosci Biobehav Rev 20: 1–25. 8622814 10.1016/0149-7634(95)00033-b

[28] Oei N, Rombouts S, Soeter R, Gerven J, Both S (2012) Dopamine modulates reward system activity during subconscious processing of sexual stimuli. Neuropsychopharmacol 37: 1729–1737. 10.1038/npp.2012.19 PMC3358742 22395731

[29] Schultz W (2006) Behavioral theories and the neurophysiology of reward. Annu Rev Psychol 57: 87–115. 16318590 10.1146/annurev.psych.56.091103.070229

[30] Lombardo MV, Ashwin E, Auyeung B, Chakrabarti B, Lai MC, et al (2012) Fetal programming effects of testosterone on the reward system and behavioral approach tendencies in humans. Biol Psychiatry 72: 839–847. 22763187 10.1016/j.biopsych.2012.05.027 PMC3485553

[31] Dewing P, Chiang CWK, Sinchak K, Sim H, Fernagut PO, et al (2006) Direct regulation of adult brain function by the male-specific factor SRY. Curr Biol 16: 415–420. 16488877 10.1016/j.cub.2006.01.017

[32] Sisk CL, Foster DL (2004) The neural basis of puberty and adolescence. Nat Neurosci 7: 1040–1047. 15452575 10.1038/nn1326

[33] Hamann S, Herman RA, Nolan CL, Wallen K (2004) Men and women differ in amygdala response to visual sexual stimuli. Nat Neurosci 7: 411–416. 15004563 10.1038/nn1208

[34] Coria-Avila GA (2012) The role of conditioning on heterosexual and homosexual partner preferences in rats. Socioaffect Neurosci Psychol 2: 17340. 24693350 10.3402/snp.v2i0.17340 PMC3960032

[35] Baumeister RF (2000) Gender differences in erotic plasticity: the female sex drive as socially flexible and responsive. Psychol Bull 126: 347–374. 10825779 10.1037/0033-2909.126.3.347

[36] Chen M, Bargh JA (1999) Consequences of automatic evaluation: Immediate behavioral predispositions to approach or avoid the stimulus. Pers Soc Psychol Bull 25: 215–224.

[37] Bancroft JHJ, Jones HG, Pullen BRA (1966) A simple transducer for measuring penile erection with comments on its use in the treatment of sexual disorder. Beh Res Ther 4: 239–241. 10.1016/0005-7967(66)90015-5 5946544

[38] Janssen E, Prause N, Geer J (2006) The sexual response system. In: Cacioppo JT, Tassinary LG, Berntson, GG (Eds.), Handbook of Psychophysiology, ed. New YorkCambridge University Press

[39] Laan E, Everaerd W, Evers A (1995) Assessment of female sexual arousal: response specificity and construct validity. Psychophysiol 32: 476–485. 10.1111/j.1469-8986.1995.tb02099.x 7568642

[40] Cousijn J, Goudriaan AE, Wiers RW (2011) Reaching out towards cannabis: approach-bias in heavy cannabis users predicts changes in cannabis use. Addict 106: 1667–1674. 10.1111/j.1360-0443.2011.03475.x PMC3178782 21518067

[41] Wiers RW, Rinck M, Kordts R, Houben K, Strack F (2010) Retraining automatic action-tendencies to approach alcohol in hazardous drinkers. Addict 105: 279–287. 10.1111/j.1360-0443.2009.02775.x 20078486

[42] Cohen J (1988) *Statistical Power Analysis for the Behavioral Sciences* (2nd ed.), New Jersey: Lawrence Erlbaum Associates.

[43] Both S, Laan E, Spiering M, Nilsson T, Oomens S, et al (2008) Appetitive and aversive classical conditioning of female sexual response. J Sex Med 5: 1386–1401. 18373525 10.1111/j.1743-6109.2008.00815.x

[44] Both S, Brauer M, Laan E (2011) Classical conditioning of sexual response in women: a replication study. J Sex Med 8: 3116–3131. 21951361 10.1111/j.1743-6109.2011.02453.x

[45] Rowland DL, Slob AK (1992) Vibrotactile stimulation enhances sexual arousal in sexually functional men: A study using concomitant measures of erection. Arch Sex Behav 21: 387–400. 1497476 10.1007/BF01542027

[46] Plaud JJ, Martini R (1999) The respondent conditioning of male sexual arousal. BehavModif 23: 254–268. 10.1177/0145445599232004 10224951

[47] Chivers ML, Seto MC, Lalumière M, Laan E, Grimbos T (2010) Agreement of self-reported and genital measures of sexual arousal in men and women: A meta-analysis. Arch Sex Behav 39: 5–56. 20049519 10.1007/s10508-009-9556-9 PMC2811244

[48] O′Donohue WT, Plaud JJ (1994) The conditioning of human sexual arousal. Arch Sex Behav 23: 321–344. 8024444 10.1007/BF01541567

[49] Everaerd W (1988) Commentary on sex research: Sex as an emotion. J Psychol Hum Sex 1: 3–15.

[50] Janssen E, Everaerd W, Spiering M, Janssen J (2000) Automatic processes and the appraisal of sexual stimuli: toward an information processing model of sexual arousal. J Sex Res 37: 8–23.

[51] Mauss IB, Robinson MD (2009) Measures of emotion: a review. Measures of emotion: A reviewMeasures of emotion: A reviewCogn Emot 23: 209–237. 10.1080/02699930802204677 PMC2756702 19809584

[52] Frijda NH (2010) Impulsive action and motivation. Biol Psychol 84: 570–579. 20064583 10.1016/j.biopsycho.2010.01.005

[53] Lang PJ (1985) The cognitive psychophysiology of emotion: Fear and anxiety. In AH Tuma & JD Maser (Eds.), Anxiety and the anxiety disorders (pp. 130–170). Hillsdale, NJ: Erlbaum.

[54] Vervliet B, Baeyens F, Van den Bergh O, Hermans D (2013) Extinction, generalization, and return of fear: A critical review of renewal research in humans. Biol Psychol 92: 51–58. 22285129 10.1016/j.biopsycho.2012.01.006

[55] Domjan M (2010) The principles of learning and behavior (6^th^ed.). Belmont, CA: Wadsworth Cengage Publishing.

[56] Hoffmann H, Goodrich D, Wilson M, Janssen E (2014) The Role of Classical Conditioning in Sexual Compulsivity: A pilot Study. Sex Addict Compulsivity 21: 75–91.

[57] Reichelt AC, Lee JLC (2013) Memory reconsolidation in aversive and appetitive settings. Front Behav Neurosci 7: 118. 24058336 10.3389/fnbeh.2013.00118 PMC3766793

[58] Ledgerwood L, Richardson R, Cranney J (2003) Effects of D-cycloserine on extinction of conditioned freezing. Behav Neurosci 117: 341–349. 12708530 10.1037/0735-7044.117.2.341

[59] Walker DL, Ressler KJ, Lu KT, Davis M (2002) Facilitation of conditioned fear extinction by systemic administration or intra-amygdala infusions of D-cycloserine as assessed with fear-potentiated startle in rats. J Neurosci 22: 2343–2351. 11896173 10.1523/JNEUROSCI.22-06-02343.2002 PMC6758267

