# Supplementary material for: Extinction and Renewal of Conditioned Sexual Responses
Source: PLoS One. 2014 Aug 29;9(8):e105955. doi: 10.1371/journal.pone.0105955 (PMC4149496; doi:10.1371/journal.pone.0105955)
Supplement: Text S1 — Supplemental Material. (DOCX) [file pone.0105955.s001.docx]

***Supplemental material***

*Design and conditioning procedure* In the preconditioning phase, the CS+ and CS- were presented for 9 seconds each. Subsequently, in the acquisition phase the contingency between CS+ and US was learned. During this phase both the CS+ and CS- were presented 10 times each and the CS+ was always followed by the US. The US was delivered for 2 seconds, starting 8 seconds after the onset of the CS+. For participants in all conditions acquisition took place in Context A. In the extinction phase, participants learned that the CS+ was no longer followed by the US. The extinction phase consisted of 10 unreinforced CS+ presentations and 10 unreinforced CS- presentations. After the extinction phase a test phase of 3 unreinforced CS+ presentations and 3 unreinforced CS- presentations was presented. For participants in the AAA condition, extinction occurred in Context A, while for participants in the ABA condition extinction took place in a different context (Context B). The switch to Context B occurred immediately after presentation of the last acquisition trial, and switching back to Context A took place immediately after presentation of the last extinction trial. Preconditioning, acquisition, extinction, and test phases were presented without interruption. There were two random CS orders for each phase; with the restriction of only two successive presentations of each CS. Half of the participants saw the pictures in order 1, the other half in order 2. During the whole procedure inter-trial intervals (ITIs) were 20, 25, or 30 seconds. The order of the length of the ITI was random, with the restriction of only two successive lengths***.***

*Stimulus Materials* A computer program timed the administration of the CS and US stimuli.

*CSs* Two neutral pictures of pictorial faces as used by Vansteenwegen et al. (2005), Effting and Kindt (2007) and Both et al. (2008, 2011). The pictures differed with regard to details of the drawings, like the nature of the hat, and the glasses the figure was wearing. Male participants were presented with cartoon-like drawings of a female character; women were presented with cartoon-like drawings of a male character. The background and size of the pictures were equal. The CSs were shown in the middle of a computer monitor, approximately 1.5 m in front of the participant. The size of the presented pictures was 14 X 21 cm.

*US* For women, a small hands-off vibrator (2 cm diameter) was used, that was built at the Department of Psychology, University of Amsterdam (Laan & van Lunsen, 2002). The vibrator was placed on the clitoris using a lycra panties that had an opening for the vaginal plethysmograph.

*Subjective Ratings* The questions were presented at the monitor 1 second following the end of picture presentation. The time the question was shown was paced by the participant’s response; the time to respond was maximally 11 seconds. When the participant answered the first question, the next question was presented after 15 seconds.

*International Index of Erectile Function (IIEF)* Male sexual functioning was assessed by the IIEF. This is a validated 15-question questionnaire that examines 4 main domains of male sexual function: erectile function (6 questions, range 0-5), orgasmic function (2 questions, range 0-5), sexual desire (2 questions, range 0-5), and intercourse satisfaction (3 questions, range 0-5). Higher scores (25-30) indicate better sexual function. Psychometric properties of the IIEF are good (Rosen et al., 1997).

*The Female Sexual Function Index (FSFI)* For women, data on sexual functioning were collected by the FSFI (Rosen et al., 2000). The FSFI consists of six subscales: desire (two items; range 1–5), arousal (four items; range 0–5), lubrication (four items; range 0–5), orgasm (three items; range 0–5), satisfaction (three items; range 0–5), and pain (three items; range 0–5). A higher score indicates better sexual functioning. The FSFI has good internal reliability and is able to differentiate between clinical samples and nondysfunctional controls (Wiegel, Meston and Rosen, 2005).

*Approach Avoidance Task* (AAT, Cousijn et al., 2011). Participants were seated in front of a computer screen with a joystick placed next to the participant’s dominant hand. During the AAT, participants pulled or pushed a joystick in response to image rotation (left or right).  Image content was irrelevant to the task: participants were instructed to pull or push the joystick in response to rotation direction, meaning some participants pushed images rotated left and pulled images rotated right, while others received opposite instructions. All images were rotated 3° left or right. Participants were randomly assigned to one of the two stimulus-response conditions. Pulling and pushing the joystick gradually increased and decreased image-size. For each assignment there were four practice trials. After the practice trials, instructions reminded participants of the stimulus-response assignment. During test trials, each CS was presented 80 times, 40 times in push- and 40 times in pull-format. Likewise, other test trials consisted 80 presentations of CS alike pictorial faces and 80 presentations of pictorial objects. The resulting 320 test trials were presented in semi-random order (at most three similar rotations and image categories in a row) and preceded by 15 practice trials with grey rectangles. The latency was recorded between picture onset and lever response. As soon as a movement with the lever was made that reached the maximum range, the picture disappeared. After an intertrial interval of 2000ms the next trail started. Data from the AAT was corrected for outliers: Response Times (RTs) below 200ms, above 2000ms and more than 3 standard deviations (SD) above and below the mean were removed for each participant. Error trials were removed. To validate the AAT, Cronbach's alpha was calculated for the two conditions with the separate bias scores for each image. Internal reliability of the CS+ bias score (Cronbach's α = .93), CS- biasscore (Cronbach's α = .92), CS-alike bias score (Cronbach's α = .95), and Neutral bias score (Cronbach's α = .92) was good. E-prime 2.0 Software (Psychology Software Tools, Inc) was used to present the stimuli.

*Exit Interview* Participants were asked about their reactions to the experimental procedure, the use of the genital device, and their evaluation of the genital vibrostimulation. Participants were asked to what extent they liked the vibrostimulation. This could be rated at a 5-point scale ranging from (1) not pleasant at all, to (5) very pleasant. Likewise, participants were asked how sexually aroused they became by the vibration. In addition, they were asked if they had had sexual fantasies during the experiment, and to what extent they had prior experience with vibrostimulation.

*Procedure* Before participation all subjects received written information, including a description of the procedure, the genital vibrostimulation, and the genital response measurement. Confidentiality, anonymity, and the opportunity to withdraw from the experiment without penalty were assured to all participants. Women were not tested during menstruation. The participants were instructed that the purpose of the experiment was to measure physiological responses to different pictures and to genital vibrostimulation. They were told that during picture viewing, brief periods of vibrostimulation would be provided. It was explained that the same pictures would be presented to them on the computer. Participants were instructed to look carefully at them, as vibrostimulation would follow some of the pictures. They were told that they should learn to predict on the basis of the pictures whether a vibration would occur or not. After placement of the vaginal probe or penile gauge and vibrator, the participant was exposed to three periods of vibrostimulation of 2 seconds, during which the participant could place the vibrator in the way it was *most sexually arousing*. It was emphasized that after final placement the position of the vibrator should not be changed during the experiment.

*Data reduction* Direct gender comparison of genital responses cannot be made because of the use of different measures to assess genital response. Therefore genital data for men and women were analyzed separately. Before analyzing the data, artifacts in penile circumference and VPA were removed. The software program VSRRP98 enables off-line graphical inspection of the data. Artifacts in the channel monitoring penile circumference and VPA can be caused by movements of the lower part of the body or by voluntary or involuntary contractions of the pelvic muscles.

The time intervals FIR, SIR and TIR are based on previous data from our laboratory (Both et al., 2008; Both et al., 2011) showing that vaginal blood engorgement is a relatively slow physiological response and that, consequently, differences in responding may not be observable during the first seconds after stimulus onset. The timeframe of SIR and TIR were included to analyze genital responding during and following (expected) US delivery.

*Results* An experimental error caused an invalid baseline value for one person, resulting in outliers on all subsequent measurements in all phases. Genital data from another participant were left out because of outliers during the whole acquisition and extinction phase. During the exit-interview this person declared to have had erotic fantasies during the experiment. Therefore, the results for women’s genital arousal are based on 60 participants (AAA= 27; ABA= 33).

**References Supplemental Material**

Both, S., Brauer, M., Laan, E. (2011). Classical conditioning of sexual response in women: a replication study. *Journal of Sexual Medicine,* 8, 3116–3131.

Both, S., Laan, E., Spiering, M., Nilsson, T., Oomens, S., Everaerd, W. (2008). Appetitive and aversive classical conditioning of female sexual response. *Journal of Sexual Medicine,* 5, 1386–1401.

Cousijn, J., Goudriaan, A. E., Wiers, R. W. (2011). Reaching out towards cannabis: approach-bias in heavy cannabis users predicts changes in cannabis use. *Addiction*, 106, 1667-1674 .

Effting, M., Kindt, M. (2007). Contextual control of human fear associations in a renewal paradigm. *Behaviour Research and Therapy*, 45, 2002-2018.

Laan, E., van Lunsen, R. H. W. (2002). *Orgasm latency, duration and quality in women: Validation of a laboratory sexual stimulation technique*. Poster presented at 28th Conference of the International Academy of Sex Research, Hamburg, Germany.

Rosen, R., Brown, C., Heiman, J., Leiblum, S., Meston, C., Shabsigh, R., Ferguson, D., D’Agostino, R. (2000). The Female Sexual Function Index (FSFI): a multidimensional self-report instrument for the assessment of female sexual function. *Journal of sex & marital Therapy*, 26, 191-208.

Rosen, R. C., Riley, A., Wagner, G., Osterloh, I. H., Kirkpatrick, J., Mishra, A., (1997). The international index of erectile function (IIEF): a multidimensional scale for assessment erectile dysfunction of erectile dysfunction. *Urology*, 49, 822-830.

Vansteenwegen, D., Hermans, D., Vervliet, B., Francken, G., Beckers, T., Baeyens, F., et al. (2005). Return of fear in a human differential conditioning paradigm caused by a return to the original acquisition context. *Behaviour Research and Therapy*, 43, 323–336.

Wiegel, M., Meston, C., & Rosen, R. (2005). The female sexual function index (FSFI): cross-validation and development of clinical cut off scores. *Journal of Sex & Marital Therapy*, 31, 1-20.
